# Phylogenetic Patterns of Colonization and Extinction in Experimentally Assembled Plant Communities

**DOI:** 10.1371/journal.pone.0019363

**Published:** 2011-05-06

**Authors:** Marc W. Cadotte, Sharon Y. Strauss

**Affiliations:** 1 Department of Biological Sciences, University of Toronto - Scarborough, Scarborough, Ontario, Canada; 2 Section of Ecology and Evolution, University of California Davis, Davis, California, United States of America; University of Zurich, Switzerland

## Abstract

**Background:**

Evolutionary history has provided insights into the assembly and functioning of plant communities, yet patterns of phylogenetic community structure have largely been based on non-dynamic observations of natural communities. We examined phylogenetic patterns of natural colonization, extinction and biomass production in experimentally assembled communities.

**Methodology/Principal Findings:**

We used plant community phylogenetic patterns two years after experimental diversity treatments (1, 2, 4, 8 or 32 species) were discontinued. We constructed a 5-gene molecular phylogeny and statistically compared relatedness of species that colonized or went extinct to remaining community members and patterns of aboveground productivity. Phylogenetic relatedness converged as species-poor plots were colonized and speciose plots experienced extinctions, but plots maintained more differences in composition than in phylogenetic diversity. Successful colonists tended to either be closely or distantly related to community residents. Extinctions did not exhibit any strong relatedness patterns. Finally, plots that increased in phylogenetic diversity also increased in community productivity, though this effect was inseparable from legume colonization, since these colonists tended to be phylogenetically distantly related.

**Conclusions:**

We found that successful non-legume colonists were typically found where close relatives already existed in the sown community; in contrast, successful legume colonists (on their own long branch in the phylogeny) resulted in plots that were colonized by distant relatives. While extinctions exhibited no pattern with respect to relatedness to sown plotmates, extinction plus colonization resulted in communities that converged to similar phylogenetic diversity values, while maintaining differences in species composition.

## Introduction

Recently, with the availability of phylogenetic information and computational tools, evolutionary history has been shown to provide insights into the assembly of plant communities [1], [2], [3], [4], [5], [6]. A number of studies have found that plant communities are often non-random assemblages of the regional plant species pool. In some cases, communities comprised species that are more closely related to each other than expected by chance [2], [4], [7], [8], while in other communities, species are more evenly distributed across the phylogenetic tree [4], [7], [8], [9]; expectations are based on random samples of species selected from a larger regional pool.

The interpretation of these phylogenetic patterns has been challenging, as phylogenetic clustering or evenness may result from a large number of ecological and evolutionary processes [5], [10], [11], [12]. For example, limiting similarity, or the concept that ecologically similar competitors compete more intensely, has been invoked as a mechanism underlying communities with an even distribution of species across the phylogenetic tree [5], [13], though this may be countered by the uneven phylogenetic distribution of competitively important traits [10]. Phylogenetic overdispersion or evenness might also reflect historical processes of speciation in sister taxa, such that sister taxa are not sympatric. Similarly, underdispersion or clustering on the tree could reflect evolutionary patterns of adaptive radiation and sympatric speciation or the presence of conserved traits of particular clades that are favored under specific abiotic conditions [5], [8], [14], [15]. In fact, numerous explanations have been proposed to explain each of these patterns. A commonality of the vast majority of these studies is that they examine communities in light of the species pool in a larger area, and implicitly invoke mechanisms operating across multiple spatial scales. For small-scale community phylogenetic patterns, those on the order of meters or smaller, we expect that local interactions could be drivers of local coexistence [16], [17].

Recent work has also shown that experimental plant assemblages with more distantly related species result in greater biomass production [18], [19]. The hypothesized mechanism explaining this pattern is that distantly related taxa are more likely to be functionally distinct or have lower niche overlap than closely related taxa [18], [19], [20]. In a North American grassland experiment, Cadotte and colleagues [19] show that phylogenetic relationships explain variation in biomass production better than a trait variation. Left unexplored is how natural community assembly processes affect the relationship between phylogeny and biomass production.

Here, we ask whether phylogenetic signal in plant community assembly can be detected in experimentally assembled plots undergoing natural colonization and extinction. We analyzed plant community compositional data originally collected as part of a BIODEPTH project in Switzerland [21]. This experiment included 64 plots planted and maintained at 1, 2, 4, 8 and 32 species between 1995 and 2000, followed by two years when natural extinction and colonization was permitted. Given that communities were created experimentally, that plots remained distinct, and that colonizations and extinctions came from a diverse pool of species, the specific questions we addressed were:

What was the phylogenetic distribution of sown species in the plots and how did this distribution change by the end of the experiment?
a) What was the overall phylogenetic distribution of colonists? ; b) Did species that successfully colonized a plot differ from those that failed to colonize in their phylogenetic distance from the plot community? We predict that species distantly related to community residents should be more likely to colonize, since they should occupy a relatively unique niche.
What were the phylogenetic distances of species that went extinct relative to those that persisted in a plot? If closely-related species have the greatest overlap in resource requirements, then we predict that species were more likely to go extinct when close relatives were present.
Were changes in biomass production at the end of the experiment related to changes in phylogenetic diversity (PD), species richness or both? We expect that communities with greater PD should be more productive, and communities that gain the most PD through colonization also show the greatest increases in productivity, since greater PD should equate with greater niche differences.

## Results

**Figure 1:**
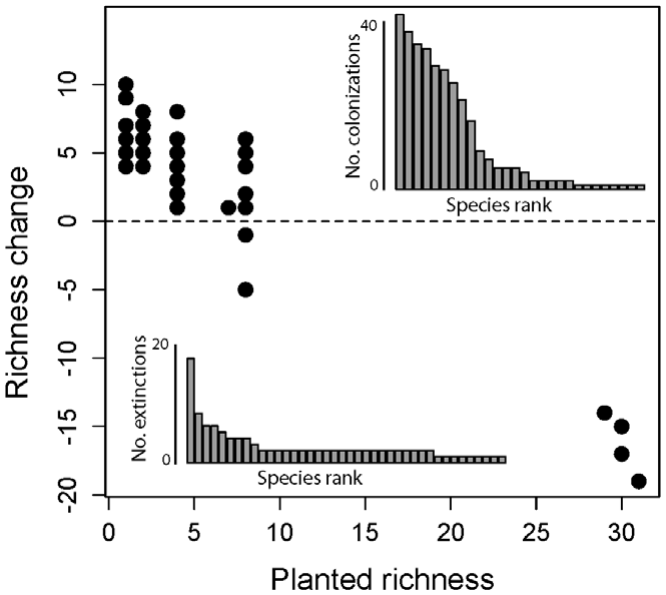
The relationship between change in the number of species in a plot and the number of species planted. Inset histograms show the number of extinction and colonization events for individual species.

By 2001, two years after experimental manipulations and weeding had ceased, plots had converged in species richness (Fig. 1). The mean number of colonizing species per community varied from 7.12 species in monocultures to 1.25 species in the most diverse communities. Colonists included species originally used in the experimental plots and those from the surrounding area and not included in a particular plot. Species extinctions per community per year increased from less than 1% of the species per plot in the years 1995–1999 (when treatments were maintained) to 10.9% in 2000 and a further 8.9% in 2001.

**Figure 2:**
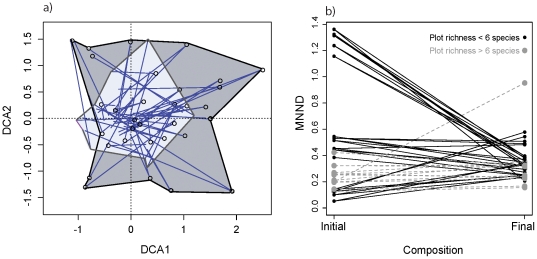
Detrended Correspondence Analysis of initial (dark grey) versus final (light grey) community compositional space (grey envelopes delineate outer envelope of starting composition of plots) (a). Circles are initial plots and lines end at final composition. Plot shows some compositional convergence, but substantial variation among plots remains. Convergence of mean nearest neighbor distances (MNND) (b), where diverse plots (i.e., with 8 or more more species—grey dashed lines) maintain MNND patterns despite species extinctions and species-poor plots (black solid lines) converge to high diversity MNND values.

Species compositions between experimental communities became more similar following cessation of weeding (mean Jaccard index in 1998 = 0.120±0.004, in 2001 = 0.378±0.004; p<0.001, see also Fig. 2a), however, there remained substantial compositional variation among plots after two years. Further, plots converged on similar mean nearest neighbor distances (MNND), with especially large decreases in initially low-richness communities as other colonists were added (Fig. 2b). This might be expected as new species colonize species-poor plots; we explore the significance and phylogenetic patterns of these colonizations below.

### Comparison 1) Phylogenetic dispersion of treatment communities

**Figure 3:**
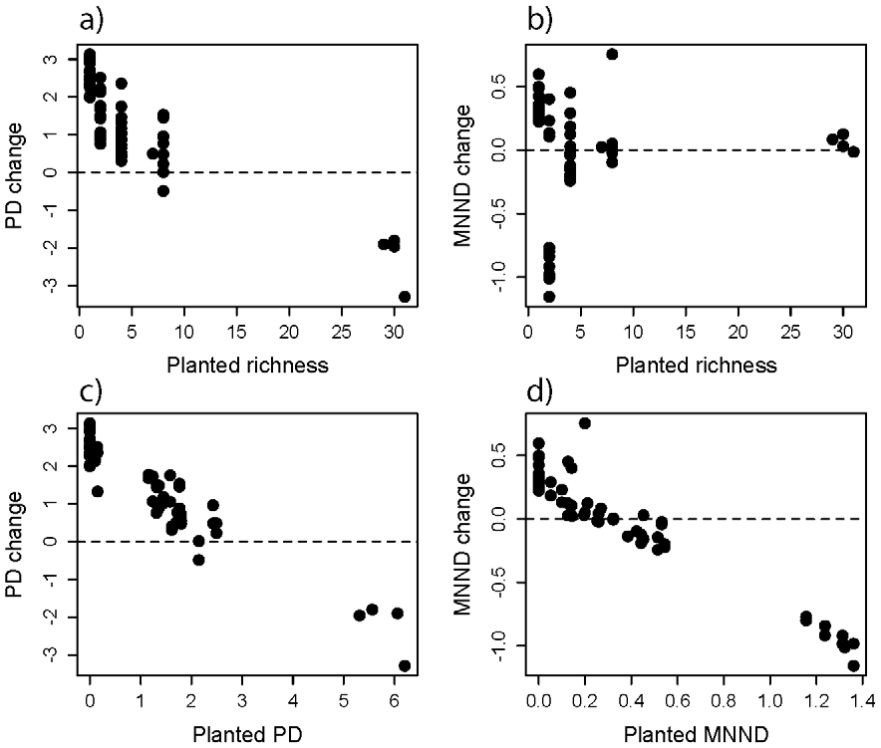
The relationship between planted diversity and diversity change in the experimental plots. Plot PD generally declined with the number of species planted (a); there was substantial change in MNND for the low diversity plots, but little change in mean MNND (b); and there appeared to be a linear relationship between planted PD and PD change (c), and between planted MNND and MNND change (d).

On average plots gained 3.51 species (sd = 6.03) and 1.28 units of phylogenetic diversity (PD) (sd = 1.30), but the 32-species treatments lost an average of 16.25 species (sd = 2.22) and 2.24 units of PD (sd = 0.705) (Fig. 3a). Interestingly, plot MNND changed very little for these diverse plots and the low diversity polycultures are centered on MNND change of zero (Fig. 3b). Further, diversity change, through colonization and extinction, resulted in an almost perfectly linear relationship between both sown PD and the change in PD (F_1,57_ = 498.8, P<0.0001, R^2^ = 0.90; Fig. 3c) and sown MNND and change in MNND (F_1,57_ = 720.7, P<0.0001, R^2^ = 0.93; Fig. 3d). It appears as though these plots are converging on PD of 2.62 (sd = 0.48) and MNND of 0.34 (sd = 0.12). Despite the remaining compositional variation among plots (Fig. 2a), these results reveal a high degree of phylogenetic convergence, where most higher-level clades are represented within communities.

### Comparison 2a) Are colonists a random subset of the species pool as a whole?

Of the 59 plots, 57 were colonized by at least one non-sown species. Species that colonized at least one plot were marginally phylogenetically clustered across the regional species pool, mean nearest neighbor distance (MNND) = 0.216, 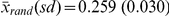, P = 0.08, suggesting some phylogenetic conservatism in colonizing ability. In particular, legumes were good colonists (*Trifolium repens* colonized 41 plots; *T. pratense*, 34 plots; and *T. flavescens*, 25 plots). Since legumes comprise a diverse clade separated by a long branch length from other groups, they can strongly influence patterns of phylogenetic dispersion of plots where they colonize. If legumes are removed from the pool of colonists, then colonists are still significantly phylogenetically clumped relative to random expectation (31 of 36 plots for which there was more than one non-legume colonist were underdispersed with 11 plots significantly so (P<0.05)); in particular, *Plantago lanceolata* (n = 37), *Arrhenatherum elatius* (n = 33), *Ranunculus acris* (n = 29) and *Taraxacum officinale* (n = 28) were conspicuous colonizers.

### 2b) Does the success of colonists depend on sown plot phylogenetic structure?

**Figure 4:**
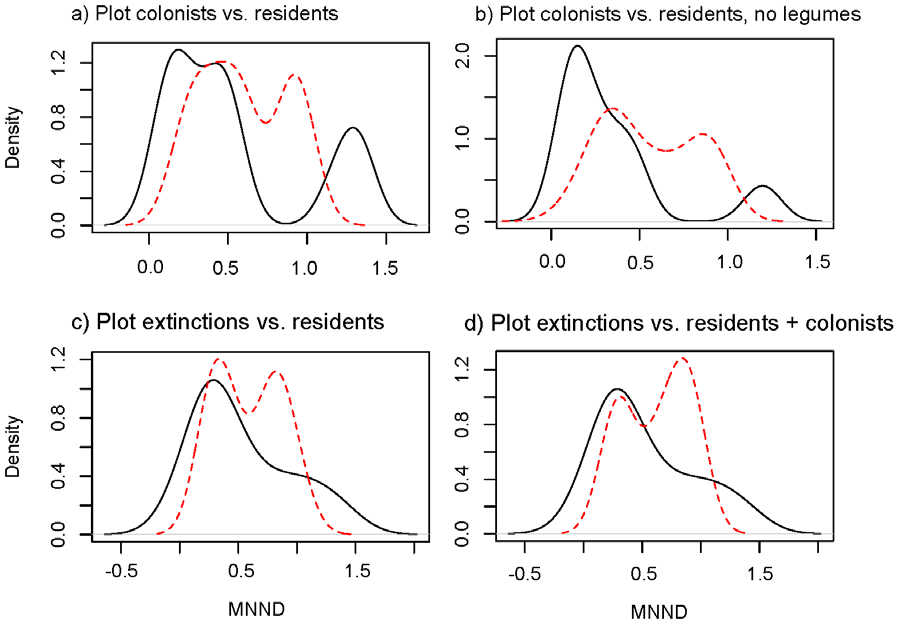
Density distributions of observed (solid line) and null (dashed line) MNND values between colonists and residents within communities (a, b) and between extinct species and residents (c, d).

Given that successful colonists came from diverse lineages phylogenetically (Asteraceae, Fabaceae, Plantaginaceae, Ranunculaceae and Poaceae), it is reasonable to ask whether the success of colonists depends on sown phylogenetic structure. In 41 colonized plots, we found 10 plots in which MNND of colonists was significantly different from random expectation, a result we interpret as exhibiting phylogenetic signal (two plots would be expected to exhibit signal by chance alone (at α = 0.05)). These results were highly affected by legumes. If legumes are included in the analysis, successful colonists tended to show a bimodal distribution of MNND values (Fig. 4a), where the majority of successful colonists either had close relatives or very distant relatives in the sown plot. For seven of the 41 plots that were colonized by multiple species, colonists were significantly phylogenetically underdispersed (P<0.05) and three were overdispersed (P<0.05) relative to sown species. When we remove the legume clade from the analysis, we still see a much larger mode of colonists that have close relatives (Fig. 4b), now with 11 of 36 plots colonized by multiple species underdispersed (P<0.05), and the second mode is greatly reduced, without any overdispersed plots. These underdispersed plots were not those found to be underdispersed initially, nor were they similar in other aspects, such as the number of species (they ranges from 7 to 25 species at the end of the study). In general, successful colonists were found where close relatives already existed.

### 3a) Are species that went extinct in plots phylogenetically clustered or overdispersed relative to species pool as a whole?

Species that went extinct in at least one plot did not exhibit any phylogenetic signal MNND = 0.215, 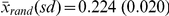, P = 0.33. Compared to the colonizers, there were not nearly as many extinction-prone species as good colonists (*Lolium perenne* went extinct 17 times and *Holcus lanatus*, 8 times). However, the most extinction-prone species were highly represented by grasses.

### 3b) Do species extinctions depend on plot phylogenetic structure?

Species extinction did not appear to be related to the phylogenetic structure of plots (Fig. 4c). In 55 plots where extinctions occurred, there was no significant relationship between extinction and MNND; in three plots, extinctions were significantly clustered relative to the species that persisted (P<0.05), and in one plot, extinction was over-dispersed (P<0.05). Again, we expect three plots to be significantly different from random by chance alone at α = 0.05, so we find that phylogenetic distance does not generally predict extinctions in plots.

Because we did not know the sequence of colonizations and extinctions in plots, we also ran the analyses with colonists as established species in the plots; adding the colonists to plot composition did not change the results (Fig. 4d). Since grasses appeared to be more likely to go extinct, we reran the analyses without grasses to see if there was a non-grass underlying pattern. The removal of grasses did not affect the results (not shown). Overall, no strong phylogenetic pattern in extinctions was evident from the data.

### 4) Changes in community productivity

**Figure 5:**
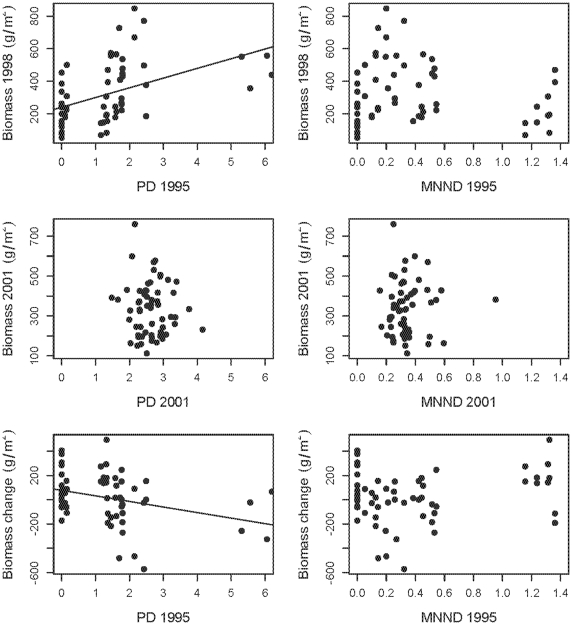
Measures of phylogenetic diversity (PD and MNND) and biomass production in 1998, 2001 and the change in biomass production. Regression line represents a statistically significant relationship.

In 1998, when the plots were still weeded, there was a significant, positive relationship between PD and biomass (F_1,57_ = 15.52, P = 0.0002, R^2^ = 0.21; Fig. 5) and between biomass and richness (F_1,57_ = 10.89, P = 0.002, R^2^ = 0.16), as also shown by Pfisterer *et al.* [21] in these plots. However, as natural colonizations and extinctions changed plot diversity, these relationships weakened, resulting in non-significant relationships between PD or richness and productivity (PD: F_1,57_ = 0.236, P = 0.629, R^2^ = 0.01; Fig. 5; richness: F_1,57_ = 0.01, P = 0.942, R^2^ = 0.00). For both years, biomass production was not significantly related to plot MNND (Fig. 5). The reason for the loss of a significant relationship between biomass and PD or richness was that plots generally converged on PD, richness and somewhat on biomass, though variation remained. The 1998 plots ranged in biomass produced from 52.4 to 847.8 g/m^2^ (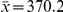, sd = 189.7 g/m^2^), while in 2001 they produced from 112.3 to 760.3 g/m^2^ (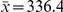, sd = 133.5 g/m^2^).

**Figure 6:**
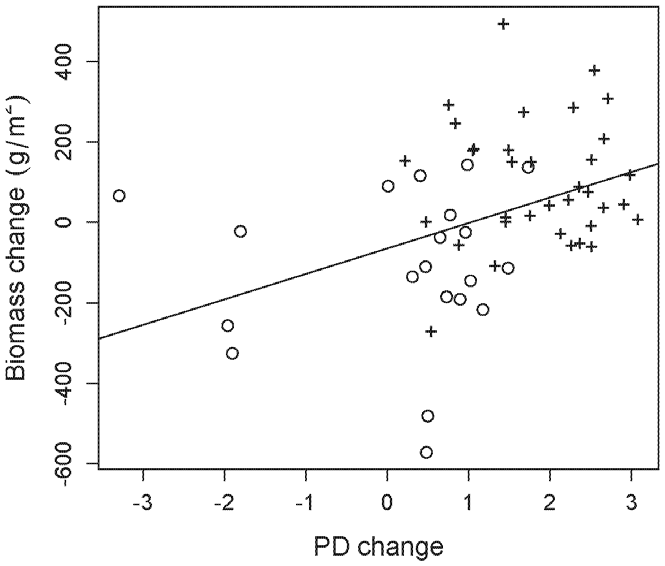
The relationships between the change in PD and change in biomass production. Open circles are plots that were initiated with a legume and ‘+’ represents plots not initiated with a legume, but were subsequently colonized by one.

Plots planted with low PD had the greatest increases in biomass production from 1998–2001, while high PD plots generally saw decreases in productivity (F_1,57_ = 6.61, P = 0.013, R^2^ = 0.10; Fig. 5). Specifically, there was a significant positive relationship between biomass change and PD change, with plots that gained PD also exhibiting increases in biomass production (F_1,57_ = 10.54, P = 0.002, R^2^ = 0.16; Fig. 6). Biomass change was only marginally related to changes in plot richness (F_1,57_ = 3.43, P = 0.07, R^2^ = 0.06), and when both PD and richness change were included in a single model explaining biomass change, PD change was a significant term while richness change was not (PD: t = 2.712, P = 0.009; richness: t = −0.828, P = 0.411). However, PD change is confounded with the addition of nitrogen fixers to plots (Fig. 6). In an ANCOVA, PD change was no longer a significant predictor of biomass change (t = 0.152, P = 0.88), while the covariate, legume presence, was significant (t = −2.372, P = 0.022). These results are consistent with earlier analyses of plots showing that legumes were both among the most common colonizers and distantly related to other species, thus plots showing the greatest increases in PD, were colonized by legumes.

## Discussion

Using data from a long-term diversity experiment in which communities were assembled at random from a regional pool, we test predictions about how the relatedness of species to established community members affects colonization success or ability to coexist, i.e., the likelihood of local extinction. While we did not find patterns of colonization and extinction that unequivocally supported a particular mechanism or pattern, we did find that phylogenetic relatedness affected community re-assembly.

Recent theoretical and a few experimental studies have shown that both evolutionary and ecological processes can give rise to assemblages built from mechanisms promoting differentiation (i.e., character displacement) and similarity (i.e., niche sharing) simultaneously [16], [22], [23], [24], [25], [26]. We found evidence that some of the colonists tended to be closely related to residents (11 plots, if legumes are excluded). Inclusion of the good-colonizing group, legumes, created a more dichotomous pattern, with colonists being either distantly or closely related to existing plot species (3 and 7 plots, respectively); this result was driven by initial experimental composition of plots and whether plots had legumes or not. Legumes themselves colonize plots with other legumes, and many plots had all three species of *Trifolium* by the end of the experiment, regardless of whether initial plot composition contained 0, 1 or 2 *Trifolium* species. This result is consistent with hints in other studies that legumes might produce facilitative relationships [e.g., 27]. The apparent coexistence of these three *Trifolium* species could be explained by the fact that since they descend from a recent common ancestor, they share traits that confer a competitive advantage in this habitat [10]. Similar fitnesses among these species means that they only require small niche differences for stable coexistence [10], [16], [28], with the proviso that our results are from only two years and thus it is impossible to infer stable coexistence.

In contrast to colonizers, species that went extinct in plots did not exhibit any strong tendency to be more or less closely related to other species planted in or colonizing a plot. A caveat to this result is that there were not many taxa in which very close relatives, like congeners, were represented in the species pool (two *Agrostis*, three *Festuca*, three *Trifolium*, which tended to co-colonize plots). So, if competitive exclusion occurs at this phylogenetic scale, then 1) we lack power to detect it at this scale and 2) the lack of congeners in communities may reflect niche-based processes that have already removed taxa that cannot coexist. Highlighting this lack of coexistence among closely-related species, grasses, as a group, were more likely to go extinct than other groups. There are two likely reasons for the susceptibility of grasses to extinction, the first is that they are, as a group, competitively inferior to other species, or that competition for limiting resources is severe within grasses resulting in high rates of competitive exclusion [29]. Our results also suggest that community assembly and change through stochastic dispersal processes were likely not an explanation for diversity changes —there was phylogenetic signal in terms of which lineages were good colonizers (legumes) and which were more prone to extinction (grasses).

Though there was some moderate compositional convergence across plots over the duration of the experiment, communities became much more convergent in the mean phylogenetic distance separating plotmates than in actual species composition. Moreover, the amount of phylogenetic diversity gained or lost was well-predicted by initial community phylogenetic diversity. However, changes in phylogenetic diversity were not substantially different than null models (Fig. S2), indicating that either average distances within plots reflect average distances from the pool of species, or that, because we lacked a true pool of potential colonists (i.e., those that could disperse into a plot but did not successfully establish), we lacked statistical power to evaluate phylogenetic change [30], [31]. In another plant community change study, Fukami and colleagues [32] compared compositional and plant trait changes over eight years and found that communities showed trait convergence but not compositional convergence [32]. Fukami et al.'s result implies that while communities vary in composition, they tend to be represented by sets of species with common suites of traits (in their case, a multitude of life-history, belowground, phenological and reproductive traits). Our results reveal that such convergence may have an evolutionary underpinning, in which plots converge on combinations of species selected from different lineages, perhaps because they occupy more diverse trait space.

While communities with greater phylogenetic diversity in 1998 were more productive, once natural assembly and coexistence mechanisms were allowed to operate, this relationship disappeared [21]. Instead, PD converged, and subsequently failed to explain variation in productivity. Other studies have shown PD–productivity relationships in experimentally-maintained plant communities [18], [19]. However, this study shows that natural community assembly may cause communities to reach convergent richness and PD levels, a result indicating that diversity effects on productivity may only be apparent during periods of biological change [21]. Pfisterer and colleagues [21] hypothesized that the convergence in richness and productivity observed in these plots was a result of species redundancy, despite divergent composition in these plots. We show that their species redundancy may be better thought of as similarity due to shared evolutionary history. Our results also reinforce the view that instead of the effects of diversity on ecosystem function, researchers should be focusing on the relationship between coexistence mechanisms and ecosystem function [33].

Our data are based on only two post-treatment years of colonization and extinction, and, while the mean number of species in plots ended up being very close to that of the mean number of species in the surrounding habitat, this was still only a two year interval. Despite the short time scale, the most diverse plots lost an average of 16.25 species, while monocultures gained an average of 7.06 species. These are dynamic and rapid changes in species numbers, and the fact that they converge on the natural mean number of species within the same habitat is striking. The convergence in species richness across plots, coupled with their convergence on the richness per meter squared in the habitat as a whole, and the convergence in phylogenetic distance separating species within plots suggest some sort of stabilizing process. Other studies have found that phylogenetic diversity in plots provides a better explanation of resilience and productivity than simply species richness [18], [19]. It would be of interest to consider dynamics over a larger time span. The immigration of early successional, post-disturbance communities is part of a larger continuum of long-term compositional changes. How do our findings compare to successional dynamics over, say, 10–20 years, or as old-fields transition into woody communities?

Another caveat to our results lies in the very strength of the study—the experimental assembly of communities. Experimental plantings may have put together some combinations species that, for whatever reasons, typically do not coexist on very local scales. Treatments were actively maintained to preserve initial composition, with 99% effectiveness, and such sustained disturbance may have resulted in communities that are more susceptible to invasion than naturally assembled communities. Despite these shortcomings, the communities that species colonized, or went extinct in, had been present for six years—with time to train soils for distinct microbial communities [34] and to deplete nutrients in species-specific ways –time to affect some of the features of habitats that others have found central to patterns of species coexistence in plant communities [35], [36].

The final caveat is the fact that we use an incomplete species pool to create our null models. There are species present at this site that were not included in the experiment and did not colonize the plots during the two years of post-treatment monitoring. Depending on the mechanisms underpinning successful colonization, the absence of non-colonizing species could bias the null models [30]. For example, if colonization ability is phylogenetically non-random, then the species pool based on successful colonist will be non-random subset of the regional phylogeny.

The assembly of natural communities is the result of a complex interplay among multiple coexistence mechanisms, and our results reveal that simple rules governing phylogenetic community patterns cannot account for species colonizations and extinctions [5], [10]. The patterns we detected were very correlated with the phylogenetic history and ecological impacts of key groups. Legumes especially were both an ecologically influential group (for plot colonization and productivity), and a well-defined clade within our species pool, having diverged from a common ancestor to sister groups more distantly than for other clades. The combination of large ecological influence and phylogenetic distinctiveness means that many overall patterns in the data set were highly influenced by legumes. Assembly and productivity patterns studied here make clear the need to understand mechanisms driving assembly and coexistence, and not just the resulting patterns of diversity.

## Materials and Methods

In spring 1995, 64 grassland communities were experimentally created in 4 m^2^ plots with 1, 2, 4, 8, or 32 plant species per m^2^ in an experimental field near Basel, Switzerland (47°N, 08°E, 439 m a.s.l.), as part of the European BIODEPTH project [for details see: 21], [37], [38]. These levels of species richness spanned the observed average species richness of 14 species/m^2^ in the surrounding grassland. Thirty-two different assemblages of species were created by constrained random sample from a pool of 48 common sympatric native local grassland species such that all polycultures contained at least one grass species. We tested for deviation from random in phylogenetic dispersion in community composition for each plot based on the 48- species pool used at the start of the experiment, given the almost-random procedure with which the species compositions were initially set up by the BIODEPTH groups. We report on this aspect in more detail below.

Each plot had a total density of 500 seedlings per m^2^ [38]; all species were perennials. Each assemblage was composed of a different set of species and all levels of diversity (combinations of species richness and number of functional groups) were represented by several different assemblages. The 32-species assemblages were planted in replicate plots in two blocks. Four legume monocultures were killed by pathogens; one polyculture was also lost, leaving a total of 59 plots that we analyzed.

The 2×2 m plots were regularly weeded to prevent invasion over four years from July 1995–September 1999. Initial weeding also eliminated species originally present in the seed bank, which consisted primarily of annuals germinating in 1995. After September 1999, treatments were no longer weeded or maintained and species were free to go extinct in, or colonize, each plot. Plots were monitored once per year for species identities and rank abundance for the next two years. Throughout the whole period (1995–2001) plots were mowed twice during the growing season (in June and September). Our analyses were all based on species composition and aboveground dry biomass of plots at the end of 2001.

### Phylogenetic analyses

Our general approach was to create phylogenetic trees for several species pools. One pool was the original set of 48 ‘internal’ experimental species; a second ‘regional’ species pool was comprised of these 48 internal species plus any other ‘external’ species that colonized any plot (an additional 12 species). We constructed a phylogeny for these species (see Fig. S1 for phylogeny and nodal support). For each of the 60 species, we searched GenBank [39] for five gene sequences commonly used in published angiosperm phylogenies: *matK*, *rbcl*, *ITS1*, *ITS2* and *5.8s*. Of the 60 species, 49 had at least one gene represented in Genbank and for the other 11 species, we used gene sequences from a congeneric relative not included in these experiments [e.g.], [ 18,19]. Collectively, the species used in this experiment represent many of the deep historical angiosperm bifurcations, relative to the number of branches connecting close relatives. Therefore, the effect on branch length estimates from using congeneric species is likely minimal, because there are relatively few polytypic genera and the effect of incorrect distance estimates at the subfamilial level is minor compared to the many large distances. We also included two representatives of early diverging angiosperm lineages as outgroup species, *Amborella trichopoda* and *Magnolia grandiflora*, which were removed prior to statistical analyses. For these species we aligned sequences using the MUSCLE algorithm [40]. We then selected best-fit maximum likelihood models of nucleotide substitution for each gene using the Akaike Information Criterion, as implemented in Modeltest [41], [42]. Using the aligned sequences and the best-fit models of nucleotide substitution, we estimated a maximum likelihood phylogeny using the PHYML algorithm with a BIONJ starting tree [43], [44]. To assess nodal support on maximum likelihood phylogenies, we report approximate Likelihood Ratio Test (aLRT) scores. The maximum likelihood tree is available in Fig. S1.

We assessed changes in phylogenetic structure using total community phylogenetic distance [PD -which differs from Faith's PD [45] by not including the root from a regional species pool] and mean nearest neighbor distance [MNND –see: 13], asking whether changes in diversity correspond with increases or decreases in species relatedness. We also examined patterns of mean pairwise distance [MPD –see: 13], but conclusions do not differ substantially from MNND and so were not reported. We compared observed PD and MNND patterns to null distributions from randomly resampling plot membership 1000 times. Null communities for experimental plots of more than one species were drawn at random from the pool of 48 species under the constraint that they had to have at least one grass species, because the vast majority of plots contain grasses and the effect of not including the long branch connect the monocots to dicots in null assemblages overshadowed real patterns. The majority of experimental communities were not significantly different than phylogenetic relationships predicted by null communities, though there was a tendency towards clustering. A total of 49 out of 59 communities were not statistically different from random expectations of mean nearest neighbour distances (MNND) (P>0.05) while 8 of 59 communities were significantly clustered and 2 were over-dispersed (P<0.05). Thus, overall, there appeared to be no consistent bias in the initial composition of plots. Moreover, inclusion or exclusion of these plots had no effect on our overall conclusions, thus we included the whole data set.

We took several strategies for analyzing and subsetting the data by classifying species into those that were planted and remained in a plot (“persist”), those that were planted into a plot in 1995 but were absent from the plot in 2001 (“extinct”) and those that were not planted in the plot but were present at the end of the experiment in 2001 (“colonize”).

Further, we asked if the observed diversity patterns and change were related to plot biomass production, using linear regressions. We also accounted for the disproportionate productivity effect of legumes (nitrogen fixers) colonization by using an analysis of covariance relating biomass change to PD change. The covariate was binary, representing either plots that initially included a legume or plots initiated without a legume and subsequently colonized by one (the 4 plots that were not initiated or colonized by a legume were excluded from this analysis). All analyses were done using R 2.9.1 (www.r-project.org) with phylogenetic manipulations and analyses done using the packages *APE* [46] and *Picante* [47], as well as functions and scripts written by the first author (http://www.utsc.utoronto.ca/~mcadotte/R_scripts.html).

## Supporting Information

Figure S1 Results of maximum likelihood phylogenetic analysis on gene sequences for species used in the experimental plots, plus two outgroup species (*Amborella trichopoda* and *Magnolia grandiflora*). On the right is the full tree showing branch lengths from the phylogenetic analysis and on the left is a rate-smoothed ultrametric tree showing nodal support.
(DOC)

Figure S2 The relationship between planted PD and the amount PD changed in plots (black dots and black dashed line) is within the possible values from 1000 random samples for each plot (grey circles and line).
(DOC)
