# Supplementary material for: Phylogenetic Patterns of Colonization and Extinction in Experimentally Assembled Plant Communities
Source: PLoS One. 2011 May 6;6(5):e19363. doi: 10.1371/journal.pone.0019363 (PMC3089622; doi:10.1371/journal.pone.0019363)
Supplement: Figure S1 — Results of maximum likelihood phylogenetic analysis on gene sequences for species used in the experimental plots, plus two outgroup species (Amborella trichopoda and Magnolia grandiflora). On the right is the full tree showing branch lengths from the phylogenetic analysis and on the left is a rate-smoothed ultrametric tree showing nodal support. (DOC) [file pone.0019363.s001.doc]

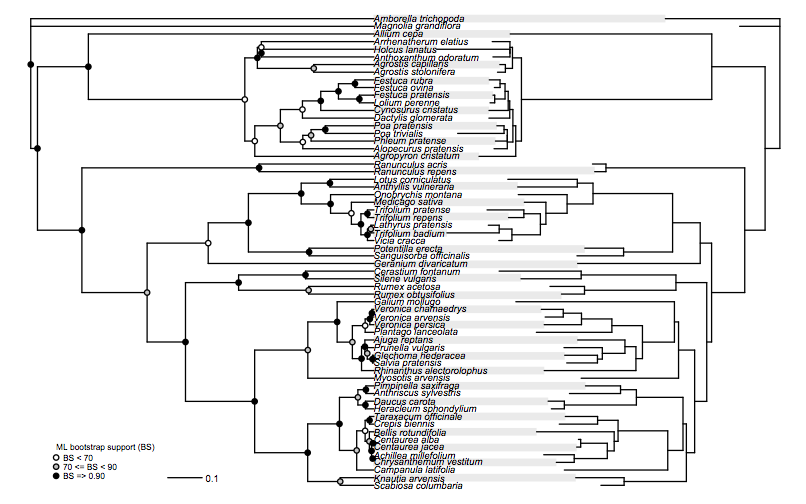


Fig. A1: Results of maximum likelihood phylogenetic analysis on gene sequences for species used in the experimental plots, plus two outgroup species (*Amborella trichopoda* and *Magnolia grandiflora*). On the right is the full tree showing branch lengths from the phylogenetic analysis and on the left is a rate-smoothed ultrametric tree showing nodal support.
