# Supplementary material for: Phylogenetic Patterns of Colonization and Extinction in Experimentally Assembled Plant Communities
Source: PLoS One. 2011 May 6;6(5):e19363. doi: 10.1371/journal.pone.0019363 (PMC3089622; doi:10.1371/journal.pone.0019363)
Supplement: Figure S2 — The relationship between planted PD and the amount PD changed in plots (black dots and black dashed line) is within the possible values from 1000 random samples for each plot (grey circles and line). (DOC) [file pone.0019363.s002.doc]

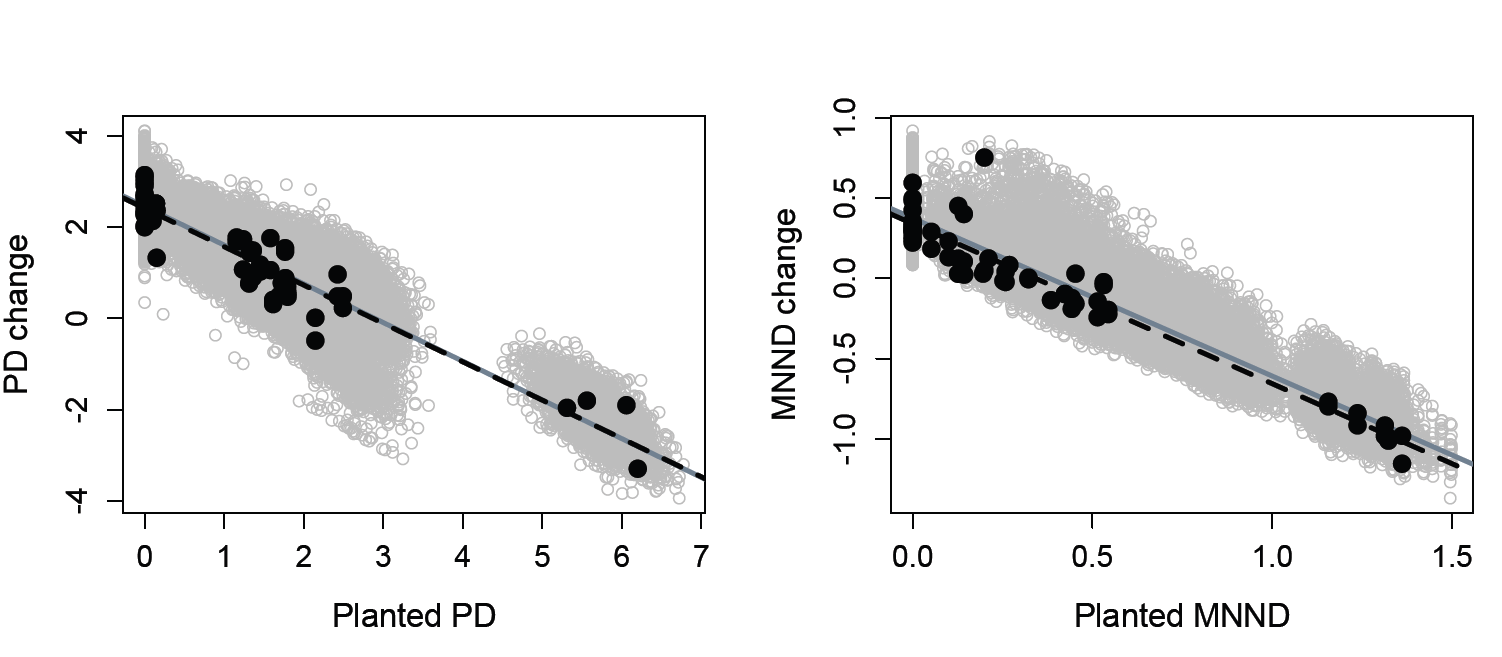


Fig. A2: The relationship between planted PD and the amount PD changed in plots (black dots and black dashed line) is within the possible values from 1000 random samples for each plot (grey circles and line).
